# Family of Two-Dimensional Transition Metal Dichlorides: Fundamental Properties, Structural Defects, and Environmental Stability

**DOI:** 10.1021/acs.jpclett.2c00367

**Published:** 2022-03-01

**Authors:** Andrey A. Kistanov, Stepan A. Shcherbinin, Romain Botella, Artur Davletshin, Wei Cao

**Affiliations:** † Nano and Molecular Systems Research Unit, University of Oulu, Oulu 90014, Finland; ‡ Peter the Great Saint Petersburg Polytechnical University, Saint Petersburg 195251, Russia; § Center for Subsurface Energy and the Environment, The University of Texas at Austin, Austin, Texas 78712, United States

## Abstract

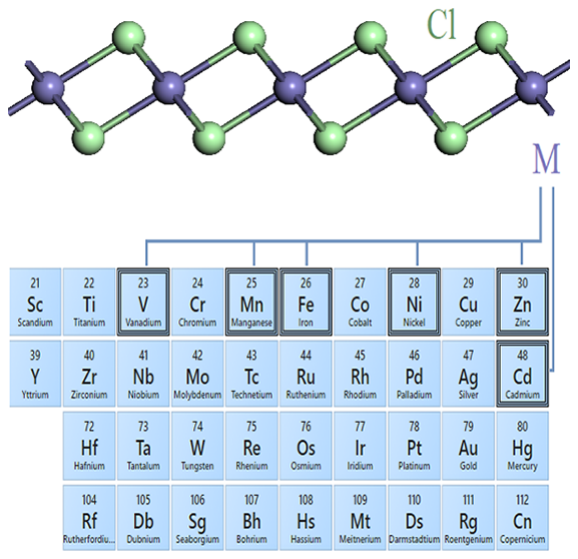

A large number of novel two-dimensional (2D) materials are constantly being discovered and deposited in databases. Consolidated implementation of machine learning algorithms and density functional theory (DFT)-based predictions have allowed the creation of several databases containing an unimaginable number of 2D samples. As the next step in this chain, the investigation leads to a comprehensive study of the functionality of the invented materials. In this work, a family of transition metal dichlorides have been screened out for systematic investigation of their structural stability, fundamental properties, structural defects, and environmental stability via DFT-based calculations. The work highlights the importance of using the potential of the invented materials and proposes a comprehensive characterization of a new family of 2D materials.

More than a decade has passed since the beginning of the era of two-dimensional (2D) materials, and the study of their discovery and applications continues unabated.^1−4^ This large family of materials presents unique properties, ranging from electronic to mechanical,^5−7^ which largely account for the high research activity in the field. Fueling the rise of 2D materials, their prediction and discovery using computational methods are revealing their wide diversity, both structural and compositional.^8−10^ There are two types of prediction strategies: combinatorial and top-down. Combinatorial approaches are based on combining an atomic composition and a crystal structure to obtain previously unexplored 2D atomic structures,^11^ while top-down approaches focus on slicing bulk materials into mono- to few-layer assemblies.^12^ These methodologies are often upscaled to high-throughput systems predicting many 2D materials, which constitutes a great achievement toward the full exploration of this part of the material space. Several imposing databases exist, filled with the results of such endeavors.^13−15^ Despite these databases being valuable warehouses of 2D materials, they can still be further supplemented with newly discovered ones.^16^ Moreover, the enormous number of existing 2D candidates lacks specificity toward prospective applications.

A promising path for identifying the application potential of the 2D species from the said databases is to study an individual family of 2D compounds with similar chemical forms.^17^ For instance, investigation of the specifics of the structure and properties of a theoretically designed family of transition metal diborides has helped to identify their application in the conversion of CO_2_.^18^ The development of transition metal carbides and nitrides has allowed selection of Ti_3_C_2_T_*x*_ monolayers possessing the highest effective Young’s modulus of ∼0.33 TPa among other solution-processed 2D materials, including graphene oxide.^19^ The criteria for picking 2D materials for most of the known applications are already well understood,^20^ with one of the most important being the environmental stability, tunability of electronic structure, and mechanical strength. An even better criterion would be the commercial availability or/and well-developed synthesis process of 2D materials, lifting technical locks impairing the investigations toward their application.

van der Waals layered transition metal dichlorides (MCl_2_) are starting to be available^21^ and can be found in databases.^13^ Therefore, they constitute very good candidates for more in-depth studies. Metal halides are commonly investigated in perovskite structures for several applications from light-emitting devices^22,23^ to nanospintronics^24^ and show tunable properties when shrinking from a bulk layered material to a monolayer.^23^ While individual transition metal halides have been studied for their unique magnetic properties,^25,26^ their environmental stability and electronic and mechanical properties have seldom been studied so far.

This work is dedicated to a DFT simulation-based systematic search of all possible existing materials in a family of 2D MCl_2_. Their structural and thermodynamical stabilities are determined by means of phonon dispersion analysis and *ab initio* molecular dynamics (AIMD) simulations. The characteristic features of screened-out 2D MCl_2_ are further analyzed to gain a comprehensive understanding of their electronic and mechanical properties. Point defect formation and surface activity of the 2D MCl_2_ toward environmental molecules are considered to facilitate their experimental observation and enlarge the area of their possible applications.

The unit cell structure of a monolayer of MCl_2_ (Figure 1) was designed on the basis of the geometry of the primitive unit cell of a monolayer of trigonal FeCl_2_ available in the 2DMatPedia database (ID dm-3574),^13^ and all transition metals (according to the periodic table) were considered as the M element. For the unit cell of each designed structure, geometry optimization was performed. The structural stability of those optimized structures was verified by calculating phonon dispersion spectra, while their thermodynamic stability was controlled by AIMD calculations.^27^ On the basis of those simulations, a stable modification of 2D MCl_2_ was proposed.

**Figure 1:**
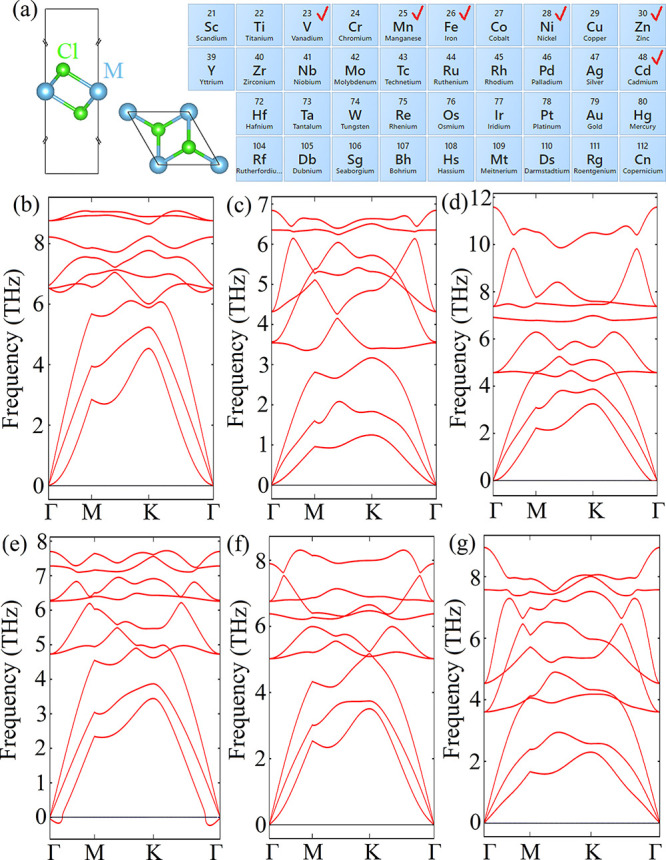
(a) Unit cell structure of 2D MCl_2_ and part of the periodic table with the marked range of the screened M elements. Phonon dispersion curves for (b) 2D FeCl_2_, (c) 2D CdCl_2_, (d) 2D MnCl_2_, (e) 2D NiCl_2_, (f) 2D VCl_2_, and (g) 2D ZnCl_2_.

The top and side views of the unit cell of 2D MCl_2_ are shown in Figure 1a. The unit cell of 2D MCl_2_ consists of one transition metal atom and two chlorine atoms. 2D MCl_2_ possesses a trigonal lattice in space group 164 *P*3̅*m*1. The kinetic stability of all possible 2D MCl_2_ forms is considered by calculating the phonon dispersion spectra along the high-symmetry directions (Γ → M → K → Γ) of the Brillouin zone. Among all 2D MCl_2_ forms, only 2D FeCl_2_, 2D CdCl_2_, 2D MnCl_2_, 2D NiCl_2_, 2D VCl_2_, and 2D ZnCl_2_ are found to be stable, as their phonon dispersion curves are positive in the whole Brillouin zone and the transverse acoustic (TA), longitudinal acoustic (LA), and out-of-plane acoustic (ZA) modes of these materials display the normal linear dispersion around the Γ point (Figure 2). Therefore, only these 2D MCl_2_ forms are further considered in this study. According to the AIMD simulations that were conducted, the listed materials also show thermal stability at 300 K (Figure S1). The structural parameters of all stable 2D MCl_2_ are listed in Table S1.

**Figure 2:**
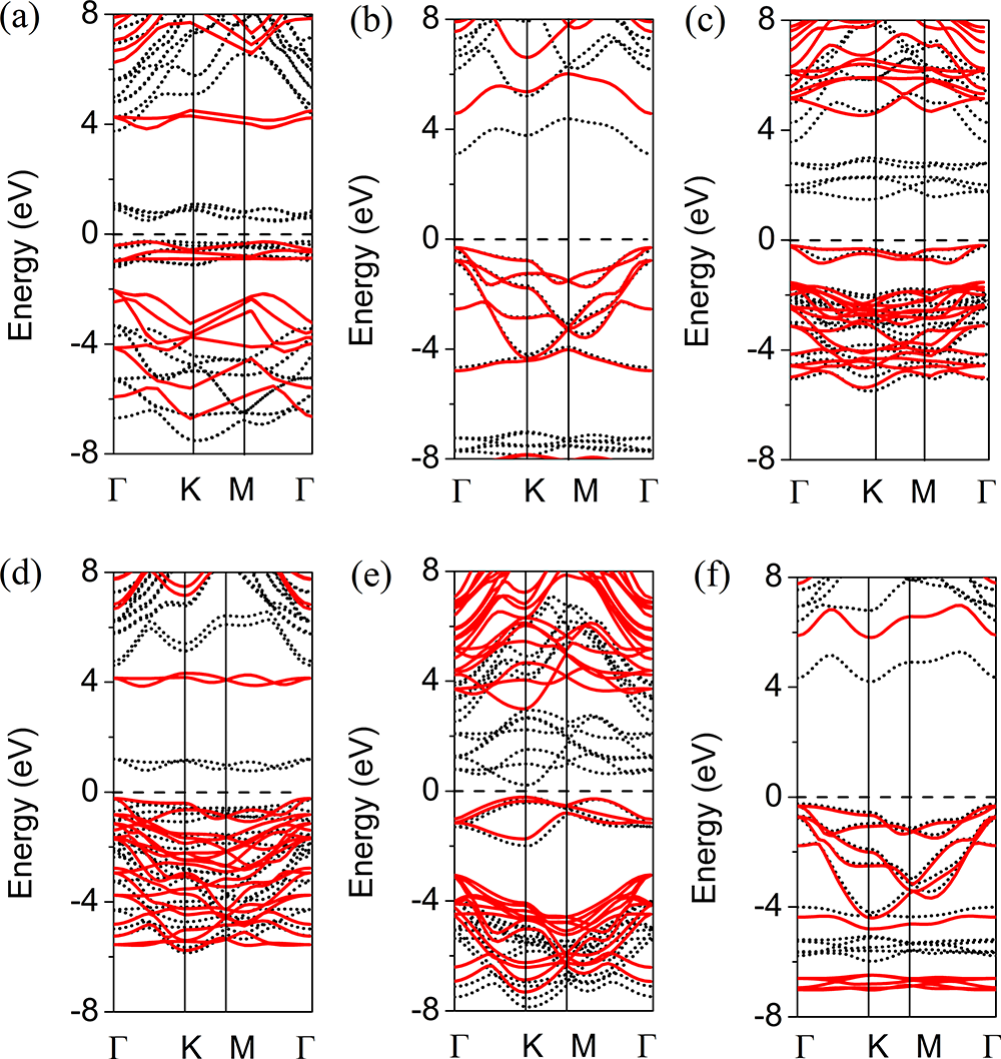
Band structures of (a) 2D FeCl_2_, (b) 2D CdCl_2_, (c) 2D MnCl_2_, (d) 2D NiCl_2_, (e) 2D VCl_2_, and (f) 2D ZnCl_2_. The black and red lines show the band structures calculated by the PBE and HSE approaches, respectively.

To evaluate possible applications of the 2D MCl_2_ forms mentioned above in electronic and straintronic devices, their electronic and mechanical properties are further considered. The band structure of 2D MCl_2_ obtained using both the Perdew–Burke–Ernzerhof (PBE) exchange-correlation functional under the generalized gradient approximation (GGA) and the Heyd–Scuseria–Ernzerhof (HSE06) functional is plotted in Figure 2, while the partial density of states (PDOS) calculated using the GGA PBE approach is plotted in Figure S2. It should be noted that the discrepancy of the band gap sizes calculated via the PBE GGA and HSE06 methods is due to lower accuracy of the PBE GGA approach, which often underestimates the width of the band gap.^28^ Therefore, the HSE06 method is expected to provide a better match with experimental results. The band gap values (*E*_g_) calculated for 2D MCl_2_ are listed in Table 1. The HSE06 approach predicts 2D FeCl_2_ is a direct band gap semiconductor with an *E*_g_ of 4.10 eV (0.85 eV according to PBE GGA). The conduction band minimum (CBM) and valence band maximum (VBM) are located between the Γ and K points. According to the PDOS plot in Figure S2a, the CBM forms because of the strong mixing of Cl p states and Fe d states and the VBM mainly consists of Fe d states. 2D CdCl_2_ is found to be a direct band gap semiconductor with an *E*_g_ of 4.88 eV (3.40 eV according to PBE GGA) and CBM and VBM located at the Γ point (Figure 2b). The PDOS plot in Figure S2b shows the CB and VB of 2D CdCl_2_ are mainly formed by Cl p states. An indirect *E*_g_ of 4.76 eV (1.60 eV according to PBE GGA) is found for 2D MnCl_2_ (Figure 2c). The CBM is located between the Γ and K points and consists of Mn d states, while the VBM is located at the Γ point and forms because of a strong mixing of Cl p states and Mn d states (Figure S2c). For 2D NiCl_2_ (Figure 2d), an indirect *E*_g_ of 4.10 eV (1.02 eV according to PBE GGA) is predicted. The CBM is located between the Γ and K points, while the VBM is located at the Γ point; both the CB and the VB are formed because of a strong mixing of Cl p states and Ni d states (Figure S2d). Figure 2e shows 2D VCl_2_ is a direct band gap semiconductor with an *E*_g_ of 3.21 eV (0.45 eV according to PBE GGA). Both the CBM and the VBM are located in the vicinity of the K points. The CB forms because of strong mixing of Cl p states and V d states, while the VB consists of only V d states (Figure S2e). An indirect *E*_g_ of 6.14 eV (4.52 eV according to PBE GGA) is found for 2D ZnCl_2_ (Figure 2f). The CBM is located in the vicinity of the K point, and CB consists of only Cl p states; the VBM is located in the vicinity of the Γ point, and the VB is formed by Cl p states and Zn d states (Figure S2f).

**Table 1:** Band Gap Sizes (*E*_g_) (HSE method), Work Functions (WF), Young’s Moduli (*E*), Shear Moduli (*G*), and Poisson’s Ratios (ν) of the Considered 2D MCl_2_ Forms

	*E*_g_ (eV)	WF (eV)	*E* (GPa)	*G* (GPa)	ν
2D FeCl_2_	4.10 (direct)	4.66	110	45	0.23
2D CdCl_2_	4.88 (direct)	7.09	36	13	0.38
2D MnCl_2_	4.76 (indirect)	6.15	47	18	0.32
2D NiCl_2_	4.10 (indirect)	6.32	107	43	0.24
2D VCl_2_	3.21 (direct)	3.90	83	33	0.29
2D ZnCl_2_	6.14 (indirect)	7.26	46	16	0.46

Table 1 also contains work function (WF) values for studied 2D MCl_2_ forms. 2D FeCl_2_ and 2D VCl_2_ possess relatively low WF values of 4.66 and 3.90 eV, respectively. In turn, 2D CdCl_2_, 2D MnCl_2_, 2D NiCl_2_, and 2D ZnCl_2_ have high WF values of 7.09, 6.15, 6.32, and 7.26 eV, respectively, which are higher than these of most 2D materials^29^ such as graphene (4.60 eV) and borophene (5.31 eV) and bulk metals^30^ such as Ni (5.23 eV) and Pt (5.65 eV). The relatively low WF of 2D FeCl_2_ and 2D VCl_2_ can be attributed to the nature of Cl atomic states around the Fermi level consisting of the out-of-plane p_*z*_ states (Figure S3a), which lie above the in-plane s–p hybridized states. As a result, the ionization of 2D FeCl_2_ and 2D VCl_2_ is comparable to that of graphene, while in 2D CdCl_2_, 2D MnCl_2_, 2D NiCl_2_, and 2D ZnCl_2_, in-plane p_*x*_ and p_*y*_ states of Cl are predominant in the vicinity of the Fermi level (Figure S3b), which explains their high WF values.

The calculated spatial dependencies of Young’s modulus, shear modulus, and Poisson’s ratio of 2D FeCl_2_ are presented in Figure 3. One can see that these quantities are direction-independent. A similar isotropic distribution of the Young’s modulus, shear modulus, and Poisson’s ratio is found for all considered 2D MCl_2_ forms (Figure S4). Therefore, each considered 2D MCl_2_ can be characterized by the in-plane Young’s modulus, shear modulus, and Poisson’s ratio. Among all considered 2D MCl_2_ forms, 2D FeCl_2_ and 2D NiCl_2_ possess the highest Young’s moduli of 110 and 107 GPa and shear moduli of 45 and 43 GPa, respectively (Table 1), which are lower than those of graphene^31^ and MoS_2_.^32^ Importantly, the Poisson’s ratio of the considered materials fell in the range of 0–0.5 (Table 1), showing their high elasticity in line with other 2D materials.^33^

**Figure 3:**
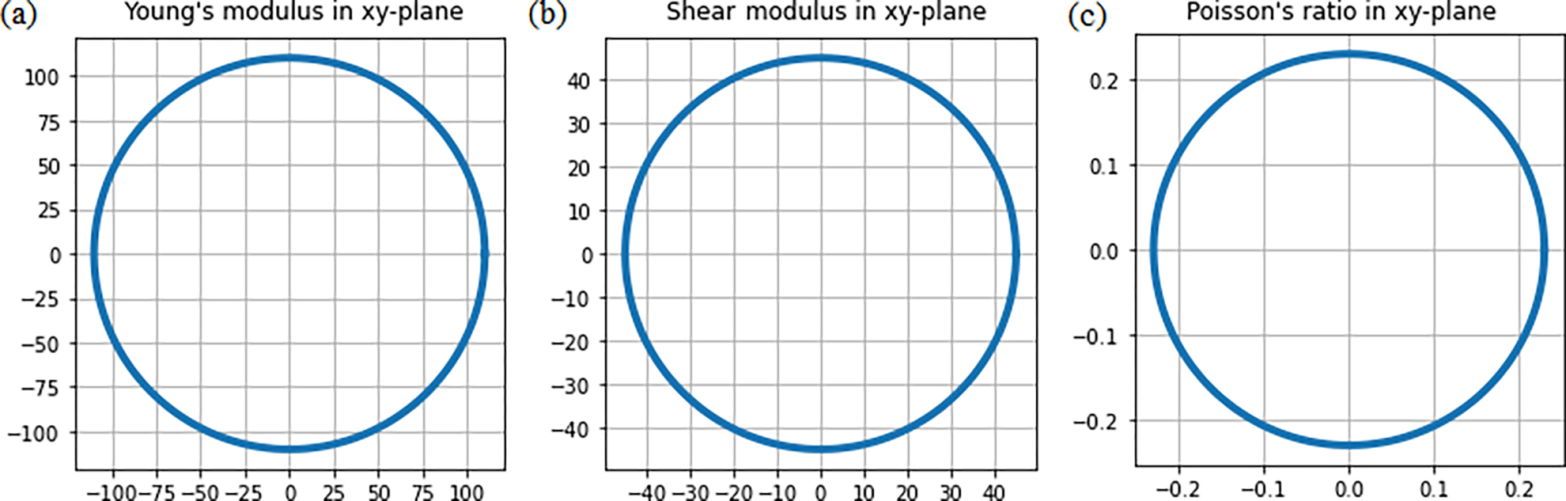
Spatial dependencies of (a) the Young’s modulus (gigapascals), (b) shear modulus (gigapascals), and (c) Poisson’s ratio for 2D FeCl_2_.

2D materials commonly host structural defects such as point defects,^34,35^ which are formed spontaneously in real systems, while their type and concentration can certainly be controlled by ion/electron irradiation or by mechanical damage of the material’s surface.^36^ Such defects may change the local structure of 2D materials and influence their properties.^37^ Therefore, a comprehensive study of the formation typical point defects in MCl_2_ is further conducted. Figure 4 (left panels) shows the atomic structure of 2D FeCl_2_ and a geometry of the most common point defects found to be stable for this structure. The stability of point defects in 2D MCl_2_ is considered in terms of their formation energy (*E*_form_). Similarly, an atomic structure of other studied 2D MCl_2_ and a geometry of the most common point defects stable in these structures are shown in Figures S5–S9 (left panels). These are a single Cl vacancy (SV_Cl_), a single M vacancy (SV_M_), a double Cl vacancy with one Cl atom on each side of the layer (DV^I^_Cl_), a double Cl vacancy on the same side of the layer (DV^II^_Cl_), and a double vacancy of one Cl atom and one M atom (DV_MCl_). It should be noted that a TMD-like structure, as in the case of 2D MCl_2_, does not contain SW defects.^38^ Two SV defects can be introduced into 2D MCl_2_ by removing the M or Cl atom from its surface, as shown in panel b or c of Figure 4 (left panels), respectively. 2D MCl_2_ can also host three various DV defects. The first is the DV^I^_Cl_ defect, which is created by removing one Cl atom from one side of the 2D MCl_2_ layer and one Cl atom from another side of the 2D MCl_2_ layer (Figure 4d, left panels). The DV^II^_Cl_ defect is created by removing two neighboring Cl atoms from one side of the 2D MCl_2_ layer (Figure 4e, left panels). The remaining DV_MCl_ defect is formed when the neighboring M atom and Cl atom are removed from the 2D MCl_2_ layer (Figure 4f, left panels).

**Figure 4:**
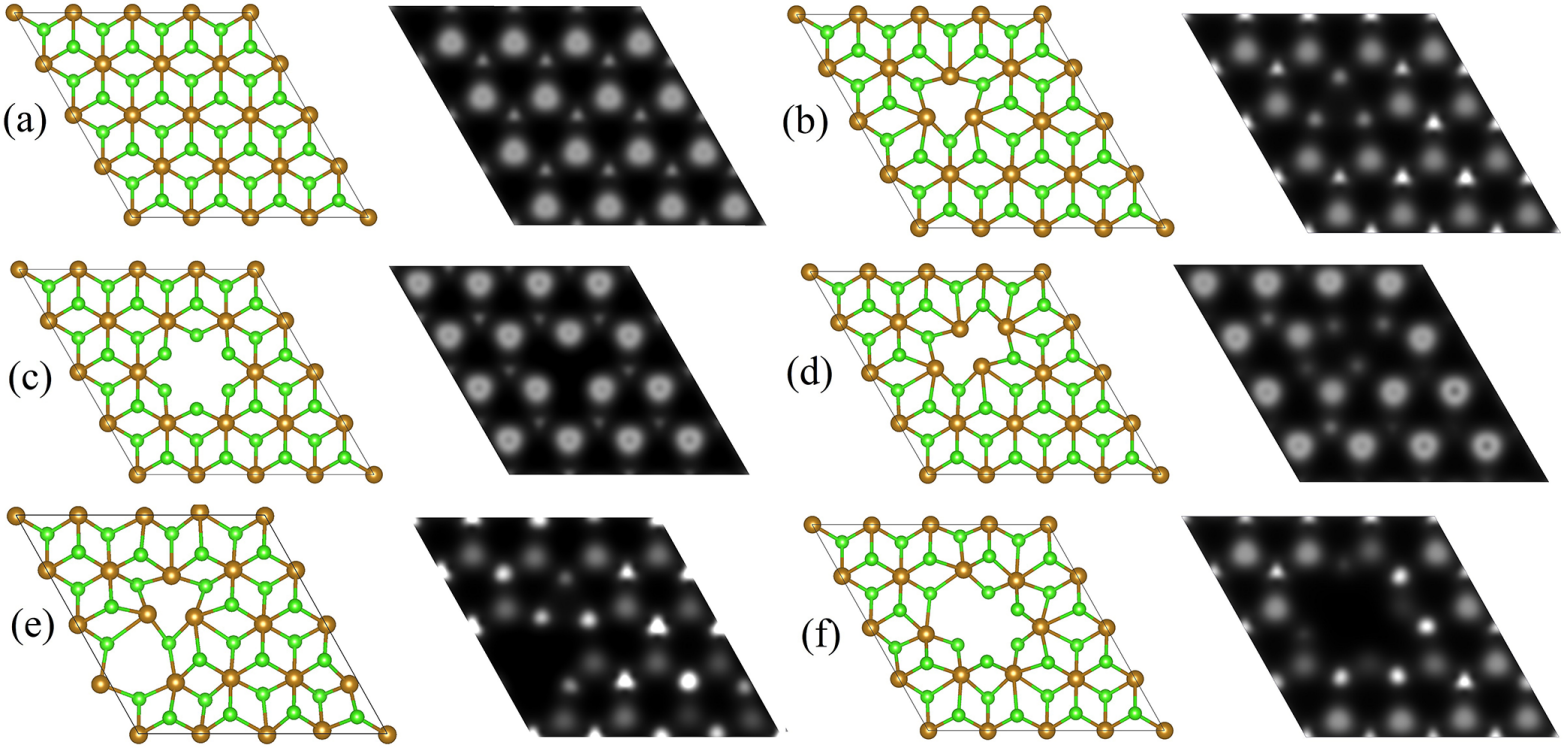
Atomic structure (left panels) and STM images at a constant height mode (the right panels) of (a) pure, (b) SV_Cl_-containing, (c) SV_M_-containing, (d) DV^I^_Cl_-containing, (e) DV^II^_Cl_-containing, and (f) DV_MCl_-containing 2D FeCl_2_.

The calculated *E*_form_ values of the considered defects in 2D MCl_2_ are listed in Table S2. According to Table S2, the SV_Cl_ defect has the lowest *E*_form_ of all of the considered 2D MCl_2_ forms. In 2D FeCl_2_, the *E*_form_ of the SV_Cl_ defect is as low as 1.04 eV, which is comparable to the *E*_form_ of SV in phosphorene (∼1–2 eV)^39^ and ∼2 times lower than that of SV in the most common 2D TMD material, MoS_2_ (∼2.12 eV).^40^ Therefore, a low *E*_form_ of the SV_Cl_ defect in 2D FeCl_2_ may lead to its instability at room temperature, similar to the case of phosphorene.^37^ Despite a low *E*_form_, the SV_Cl_ defect in 2D FeCl_2_ possesses high stability, which is confirmed by AIMD simulations at room temperature for 3 ps (Movie 1). The *E*_form_ of SV_Cl_ of 3.23 eV in 2D NiCl_2_ is higher than that of the SV defect in MoS_2_ while still significantly lower than the *E*_form_ of SV in graphene (7.5 eV).^41^ For 2D CdCl_2_, 2D MnCl_2_, 2D VCl_2_, and 2D ZnCl_2_, the *E*_form_ values of the SV_Cl_ defect are 4.75, 4.56, 5.14, and 5.02 eV, respectively, that are significantly higher than that of the SV defect in MoS_2_ but still lower than that of the SV defect in graphene. It should be noted that DV defects in 2D MCl_2_ (except 2D FeCl_2_) have *E*_form_ values (∼7–10 eV) higher than that of DV defects in most common 2D materials, including graphene (∼8 eV)^41^ and MoS_2_ (∼4 eV).^42^

A remarkable difference in the *E*_form_ values of SV defects in 2D MCl_2_ can be attributed to the difference in the electronegativity of M elements compared to that of Cl.^43^ It is known that, if the difference in electronegativity of a bonded metal and nonmetal is ≳1.5, a compound is expected to be ionic, while a covalent type of bonding is expected when the electronegativity of a bonded metal and non-metal is ≲1.5. Therefore, the bonds in 2D FeCl_2_ and 2D NiCl_2_ are expected to be covalent in nature, as the difference in the electronegativity of Cl (3.0) and both Fe (1.8) and Ni (1.9) is ≳1.5. On the contrary, the difference in the electronegativity of Cl (3.0) and Cd (1.7), Mn (1.5), V (1.6), and Zn (1.6) is close to ∼1.5, which suggests the existence of ionic bonds between these compounds. To support this conclusion, the electron localization function for 2D FeCl_2_ and 2D ZnCl_2_ is analyzed.^44−47^ In the case of 2D FeCl_2_ (Figure S5a), the electron localization isobserved on Fe atoms and partially on the Fe–Cl bond, which confirm the existence of an ionocovalent type of bonding in 2D FeCl_2_. In the case of 2D ZnCl_2_ (Figure S5b), the electron localization basin is spherical and completely migrates to the Zn atom so that basins are all surrounding the respective cores, suggesting an ionic bond in 2D ZnCl_2_. Therefore, strong ionic bonds in 2D CdCl_2_, 2D MnCl_2_, 2D VCl_2_, and 2D ZnCl_2_ can explain their high stability against the formation of most point defects compared to 2D FeCl_2_, 2D NiCl_2_, and common 2D materials.

To facilitate the experimental identification of point defects in 2D MCl_2_, simulated scanning tunneling microscopy (STM) images are obtained for perfect and defect-containing 2D MCl_2_. A constant height mode characterization method is used in all cases. The STM images of the perfect and defect-containing 2D FeCl_2_ are presented in Figure 4 (right panels), while the STM images of defects for other studied 2D MCl_2_ forms are shown in Figures S6–S10. Defects are easy to recognize at STM images and correlate well with their atomic structures. For instance, the STM image at Figure 4b (right panel) clearly reflects the SV_Cl_ defect with a triangle formed of three bright spots characterizing three Cl atoms inside of which one Cl is missing. Similarly, Figure 4c (right panel) shows the SV_Fe_ defect presented by a triangle formed of three large bright spots characterizing three Cl atoms and a pentagon formed of five small bright spots characterizing five Fe atoms inside of which one Fe is missing. The most complicated task is to differentiate the DV^I^_Cl_ defect, which may be confused with the SV_Cl_ defect. However, as opposed to the SV_Cl_ defect, in the case of DV^I^_Cl_, four small bright spots in the form of a parallelogram reflecting four Fe atoms with one missing Cl atom inside are clearly visible (Figure 4d, right panel). The STM image of the DV^II^_Cl_ defect is presented in Figure 4e (right panel); there the formation of the triangle of three small bright spots as three Fe atoms are shifted due to the absence of two neighboring Cl atoms (two dim spots are absent) is seen. The DV_FeCl_ defect is visible in the STM image (Figure 4f, right panel) as there one small (Fe atom) and one large (Cl atom) bright spot are clearly missing.

It is well-known that 2D materials are highly sensitive to the environmental conditions.^48−50^ To determine the behavior of 2D MCl_2_ under environmental conditions, particularly in the presence of moisture, their interaction with H_2_O and O_2_ molecules is considered. All possible adsorbing configurations of H_2_O and O_2_ on studied 2D MCl_2_ forms are considered. The determined lowest-energy configurations, together with adsorption energy *E*_a_ for the H_2_O and O_2_ molecules on 2D MCl_2_, are presented in Figures S11 and S12. In cases of 2D FeCl_2_, 2D CdCl_2_, 2D MnCl_2_, 2D NiCl_2_, and 2D ZnCl_2_, the H_2_O and O_2_ molecules are located above the metal site with the O atom directed to the surface. The *E*_a_ of H_2_O and O_2_ on these materials is comparably high (Figures S11 and S12) and is comparable to that of H_2_O and O_2_ on other common 2D materials (Table S3), such as graphene,^51^ 2D pnictogens,^49,52^ and a family of 2D phosphorus carbides.^53^ 2D VCl_2_ stands out from its counterparts as the H_2_O and O_2_ molecules have a 2 times lower *E*_a_ on its surface and the Cl atom is located with both H atoms (in case of H_2_O) and the O atom (in case of O_2_) tending to two other Cl atoms at the surface. The calculated *E*_a_ of H_2_O and O_2_ on 2D MCl_2_ is comparably high; therefore, these materials are supposed to be environmentally stable. This is also confirmed via AIMD simulations in which a weak interaction of the 2D FeCl_2_ surfaces with H_2_O (Movie 2) and O_2_ (Movie 3) at room temperature is shown. It should be noted that metal chlorines usually possess strong electron donating and/or accepting abilities, making these materials active for adsorbents.^54^ One of the reasons for that can be their constituent elements with weak or strong electronegativities or high ionicity. Another reason can be a comparably low *E*_form_ of defects in 2D MCl_2_ forms, which can also affect their stability. For instance, it is found that the *E*_a_ of H_2_O on 2D MCl_2_ decreases by 6-fold (from −0.12 to −0.66 eV) in the presence of a SV_Cl_ defect compared to that of H_2_O on pure 2D MCl_2_. On the contrary, as it has been shown for metal (hydr)oxides, the adsorption of various species on metal chloride surfaces under moisture and/or water-saturated conditions can be hindered.^55^ Therefore, oxygen-passivated and water-saturated metal-containing materials can exhibit higher stability to adsorbents. We can conclude that despite the fact that the studied 2D MCl_2_ forms are found to be stable under environmental conditions, their stability may be affected by many factors, such as surface hydration and defect formation.

In summary, in this work following a sequential search over existing databases of 2D materials and the subsequent systematic screening of possible atomic combinations, a new family of 2D MCl_2_ forms, consisting of 2D FeCl_2_, 2D CdCl_2_, 2D MnCl_2_, 2D NiCl_2_, 2D VCl_2_, and 2D ZnCl_2_, has been identified. DFT-based simulation has been implemented to prove the structural stability of the screened-out materials and systematically study their fundamental properties and structural changes under certain conditions, such as the presence of point defects and a moisture environment. 2D MCl_2_ forms, due to their electronic and mechanical properties, are shown to be versatile candidates in the semiconductor industry, while the defect-related and ambient stabilities demonstrate their durability and the feasibility of their manipulation. In particular, 2D MnCl_2_, 2D NiCl_2_, and 2D ZnCl_2_ due to their high WF values can be used in carrier transport nanoelectronic devices, while a high Young’s modulus and a shear modulus of 2D FeCl_2_ and 2D NiCl_2_ make them good candidates for straintronic devices.^56^ This work highlights the importance of the developing databases of 2D materials and the need for a deep investigation and characterization of materials available in the existing databases.

## Computational Methods

All calculations were performed using the plane-wave method as implemented in the Vienna Ab initio Simulation Package (VASP).^57^ The PBE exchange-correlation functional under the GGA^58^ was used for the geometry optimization calculations, while the electronic structure calculations were supplemented with the HSE functional.^59^ The considered supercells of 2D MCl_2_ were composed of 4 × 4 × 1 unit cells (16 M and 32 Cl atoms) to avoid nonphysical interactions between periodic images while keeping the computational cost affordable. The optimization was stopped once the atomic forces and total energy values were <10^–4^ eV/Å and <10^–8^ eV, respectively. The first Brillouin zone was sampled with a 15 × 15 × 1 k-mesh grid for the unit cell and a 3 × 3 × 1 k-mesh grid for the 4 × 4 × 1 supercell. The kinetic energy cutoff was set at 520 eV. The periodic boundary conditions were applied for the two in-plane transverse directions, while a vacuum space of 20 Å was introduced in the direction perpendicular to the surface plane. Under such conditions, the concentrations of SV and DV defects were 2.08% (one M/Cl atom per 48 atoms) and 4.17% (two M/Cl atoms per 48 atoms), respectively.

The finite displacement approach as implemented in the Phonopy code^60^ was used to simulate phonon dispersion spectra. The AIMD simulation lasts for 5 ps with a time step of 1.0 fs, and a temperature of 300 K was controlled by a Nose–Hoover thermostat. STM images were simulated via the Tersoff–Hamann approach.^61^

The stress–strain relation was used to calculate the components of the stiffness matrix for the considered structures.^62^ For these calculations, approximate interlayer distances were used. The interlayer distance was considered to be the distance at which the force of action between the layers becomes <0.01 eV/A. On the basis of the obtained stiffness matrix, the Young’s modulus, shear modulus, and Poisson’s ratio were calculated and the directional dependencies of these quantities were defined using ELATE software for the analysis of elastic tensors.^63^

The stability of the considered point defects in 2D MCl_2_ was considered on the basis of their formation energy (*E*_form_), which was calculated as
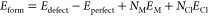
1where *E*_defect_ and *E*_perfect_ are the total energies of perfect and defect-containing 2D MCl_2_, respectively, *E*_M_ and *E*_Cl_ are the energies of a single transition metal and chlorine atom, respectively, and *N*_M_ and *N*_Cl_ correspond to the number of the removed transition metal and chlorine atoms, respectively.

## Supporting Information Available

Structural parameters of 2D MCl_2_ (Table S1), thermal stability of 2D MCl_2_ (Figure S1), PDOS of 2D MCl_2_ (Figures S2 and S3), Young’s moduli, shear moduli, and Poisson’s ratios of 2D MCl_2_ (Figure S4), *E*_form_ values of point defects in 2D MCl_2_ (Table S2), electron localization function for 2D FeCl_2_ and 2D ZnCl_2_ (Figure S5), STM images of point defects in 2D MCl_2_ (Figures S6–S10), and absorption of H_2_O and O_2_ on 2D MCl_2_ (Figures S11 and S12 and Table S3) (PDF)
Stability of the SV in 2D FeCl_2_ (MP4)
H_2_O absorption on 2D MCl_2_ (MP4)
O_2_ absorption on 2D MCl_2_ (MP4)
2D FeCl_2_ primitive cell (CIF)
2D CdCl_2_ primitive cell (CIF)
2D MnCl_2_ primitive cell (CIF)
2D NiCl_2_ primitive cell (CIF)
2D VCl_2_ primitive cell (CIF)
2D ZnCl_2_ primitive cell (CIF)

## Supplementary Material

jz2c00367_si_004.mp4

jz2c00367_si_005.cif

jz2c00367_si_006.cif

jz2c00367_si_007.cif

jz2c00367_si_008.cif

jz2c00367_si_009.cif

jz2c00367_si_010.cif
