# Supplementary material for: Family of Two-Dimensional Transition Metal Dichlorides: Fundamental Properties, Structural Defects, and Environmental Stability
Source: J Phys Chem Lett. 2022 Mar 1;13(9):2165–72. doi: 10.1021/acs.jpclett.2c00367 (PMC8919257; doi:10.1021/acs.jpclett.2c00367)
Supplement: Supplementary file 1 — jz2c00367_si_001.pdf [file jz2c00367_si_001.pdf]

Supplementary Information

Family of Two-Dimensional Transition Metal Dichloride's:

Fundamental Properties, Structural Defects, and Environmental Stability

Andrey A. Kistanov[1,*], Stepan A. Shcherbinin[2], Romain Botella[1], Artur Davletshin[3], Wei Cao[1]

[1]Nano and Molecular Systems Research Unit, University of Oulu, Oulu 90014, Finland

[2]Peter the Great Saint Petersburg Polytechnical University, Saint Petersburg 195251, Russia

[3]Center for Subsurface Energy and the Environment, The University of Texas at Austin, Austin, Texas 78712, United States

*Corresponding Author: andrey.kistanov@oulu.fi (AAK)

Section 1. Structural parameters of 2D MCl_2

Table S1. Structural parameters of 2D MCl_2

	a, Å	*b*, Å	*α*, °	*β*, °	*γ*, °	*M-Cl bond length*, Å	*Interlayer distance*, Å
2D $FeCl_2$	3.41	3.41	90	90	120	2.30	6.5
2D $CdCl_2$	3.91	3.91	90	90	120	2.68	6.6
2D $MnCl_2$	3.38	3.38	90	90	120	2.34	6.7
2D $NiCl_2$	3.44	3.44	90	90	120	2.38	6.5
2D VCl_2	3.22	3.22	90	90	120	2.41	6.9
2D $ZnCl_2$	3.60	3.60	90	90	120	2.49	6.6

Section 2. Thermal stability of 2D MCl_2

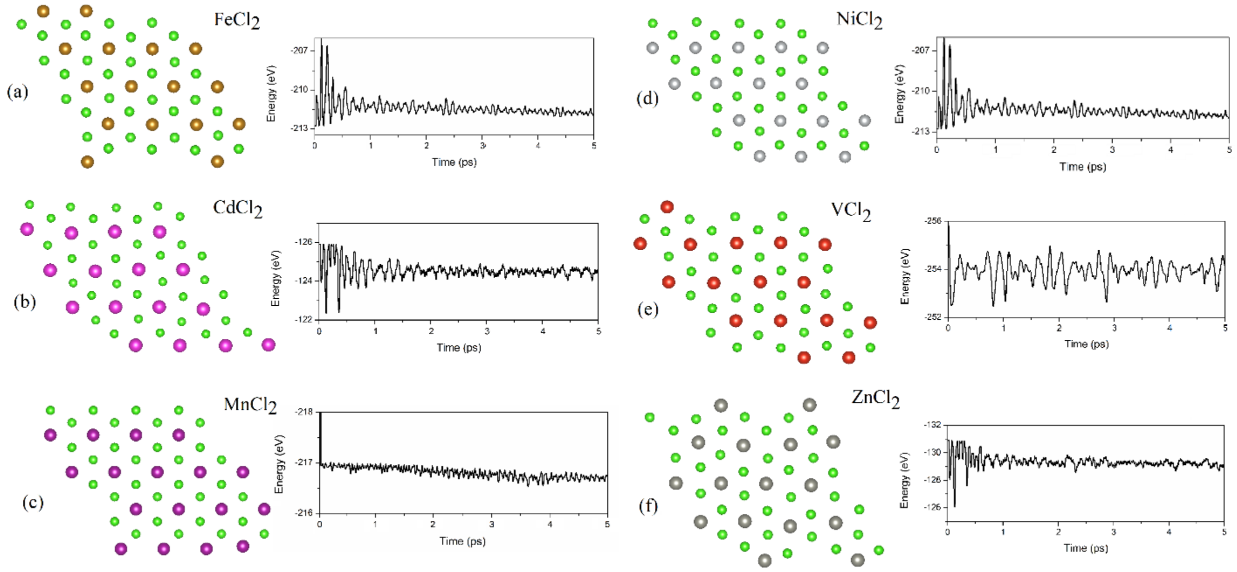

Figure S1. Atomic structure and total energy fluctuation obtained from AIMD simulations conducted at 300 K for the time of 5 ps for (a) 2D $FeCl_2$, (b) 2D $CdCl_2$, (c) 2D $MnCl_2$, (d) 2D $NiCl_2$, (e) 2D VCl_2, (f) 2D $ZnCl_2$.

Section 3. PDOS of 2D MCl_2

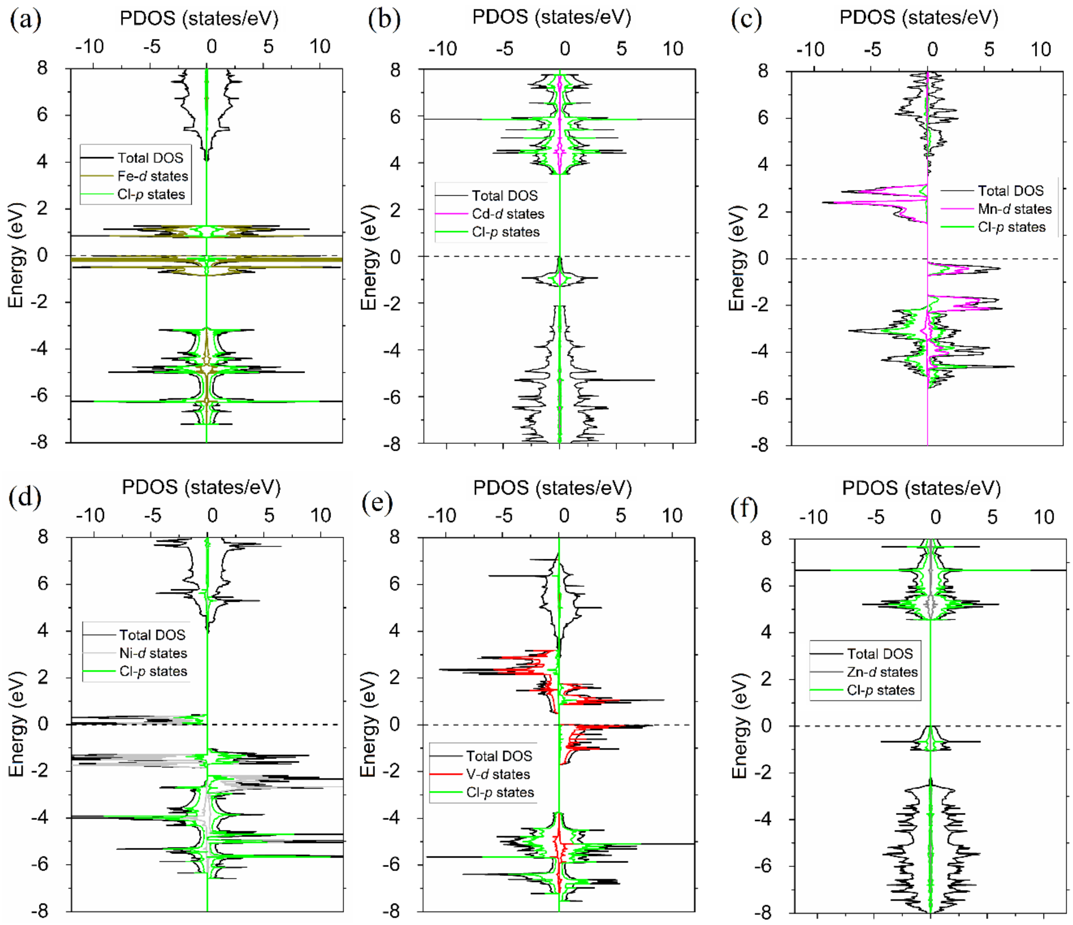

Figure S2. PDOS for (a) 2D $FeCl_2$, (b) 2D $CdCl_2$, (c) 2D $MnCl_2$, (d) 2D $NiCl_2$, (e) 2D VCl_2, and (f) 2D $ZnCl_2$ calculated by the PBE GGA approach.

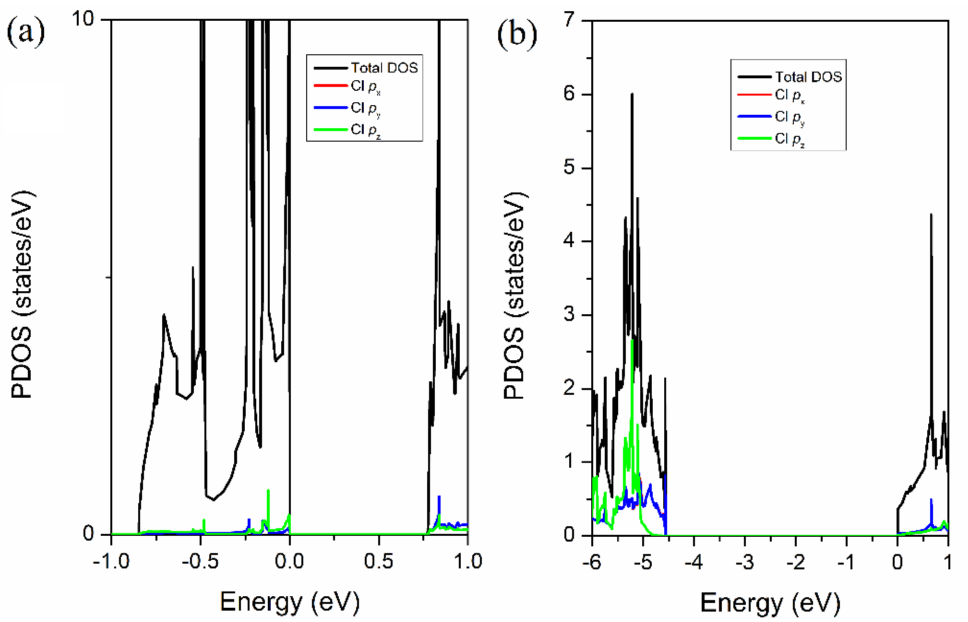

Figure S3. PDOS for (a) 2D $FeCl_2$ and (b) 2D $ZnCl_2$ calculated by the PBE GGA approach.

Section 5. Spatial dependencies of Young’s modulus, shear modulus, and Poisson’s ratio of 2D MCl_2

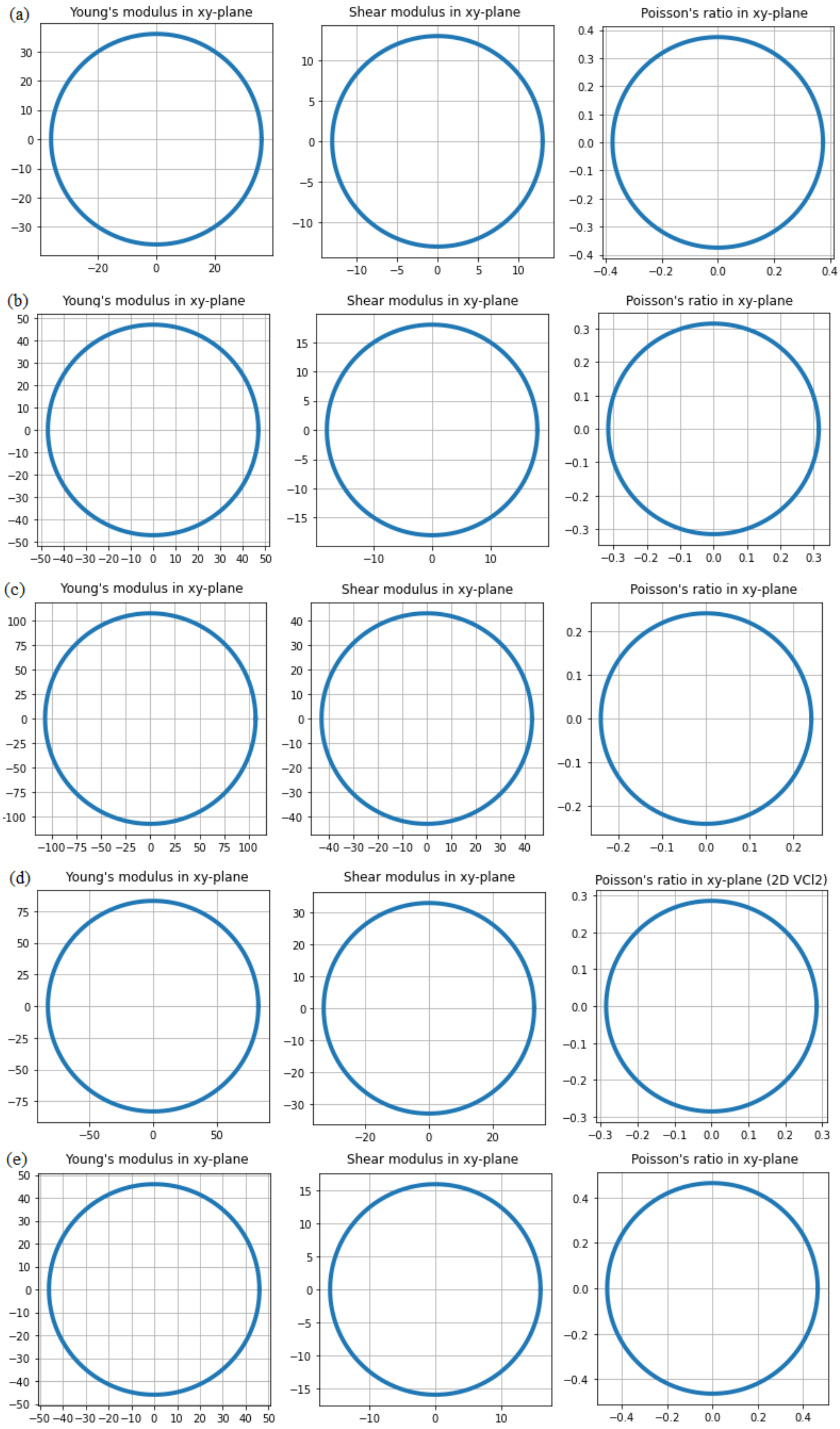

Figure S4. Spatial dependencies of Young’s modulus (in GPa), shear modulus (in GPa), and Poisson’s ratio for (a) 2D $CdCl_2$, (b) 2D $MnCl_2$, (c) 2D $NiCl_2$, (d) 2D VCl_2, and (e) 2D $ZnCl_2$.

Section 6. Point defects in 2D MCl_2

Table S2. E_{form}, eV of point defects in 2DMCl_2.

	SV_{Cl}	SV_M	DV^I_{Cl}	DV^{II}_{Cl}	DV_{MCl}
2D $FeCl_2$	**1.04**	3.63	4.01	3.17	1.51
2D $CdCl_2$	**4.75**	6.68	7.21	7.91	7.54
2D $MnCl_2$	**4.56**	7.12	9.33	9.37	8.92
2D $NiCl_2$	**3.23**	7.37	6.75	6.51	9.61
2D VCl_2	**5.14**	5.47	10.36	10.26	10.01
2D $ZnCl_2$	**5.02**	6.64	7.41	8.18	7.51

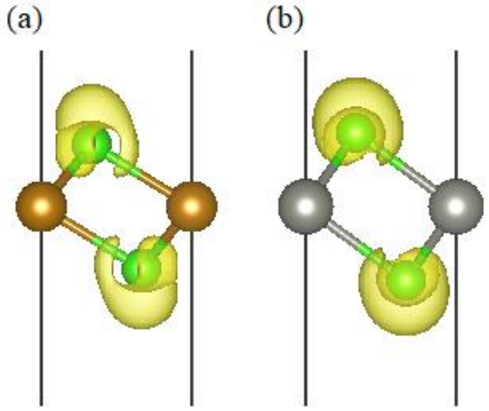

Figure S5. Electron localization function for (a) 2D $FeCl_2$ and (b) 2D $ZnCl_2$.

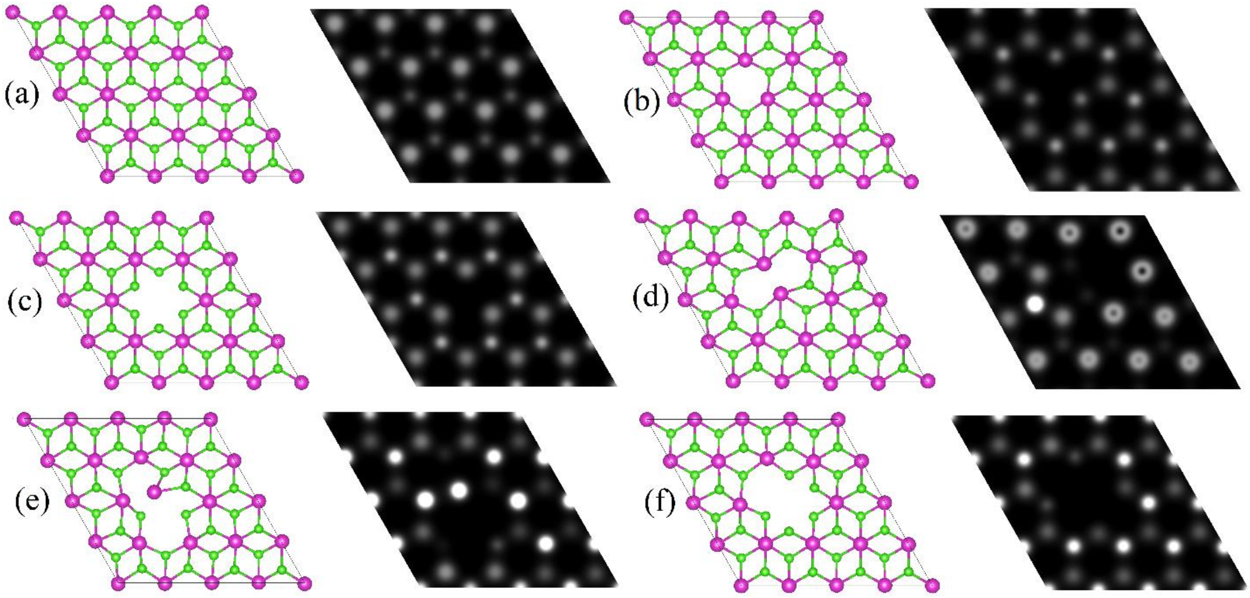

Figure S6. Atomic structure (the left panels) and STM images at a constant height mode (the right panels) of (a) pure, (b) SV_{Cl}-containing, (c) SV_M-containing, (d) DV^I_{Cl}-containing, (e) DV^{II}_{Cl}-containing, and (f) DV_{MCl}-containing 2D $CdCl_2$.

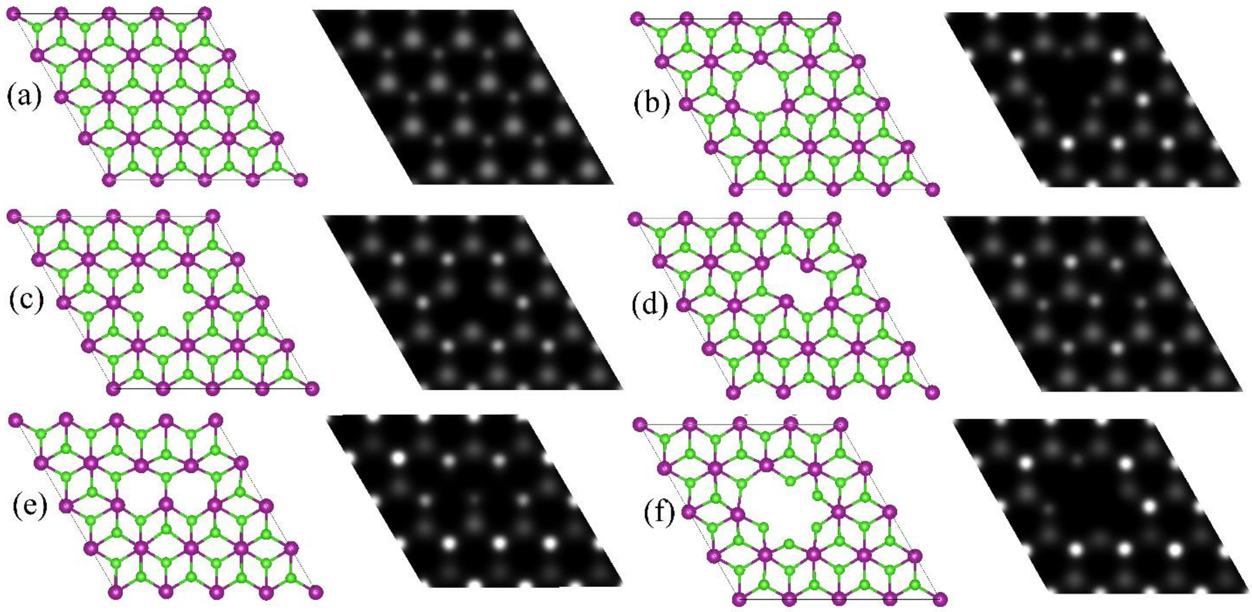

Figure S7. Atomic structure (the left panels) and STM images at a constant height mode (the right panels) of (a) pure, (b) SV_{Cl}-containing, (c) SV_{M}-containing, (d) DV^{I}_{Cl}-containing, (e) DV^{II}_{Cl}-containing, and (f) DV_{MCl}-containing 2D $MnCl_2$.

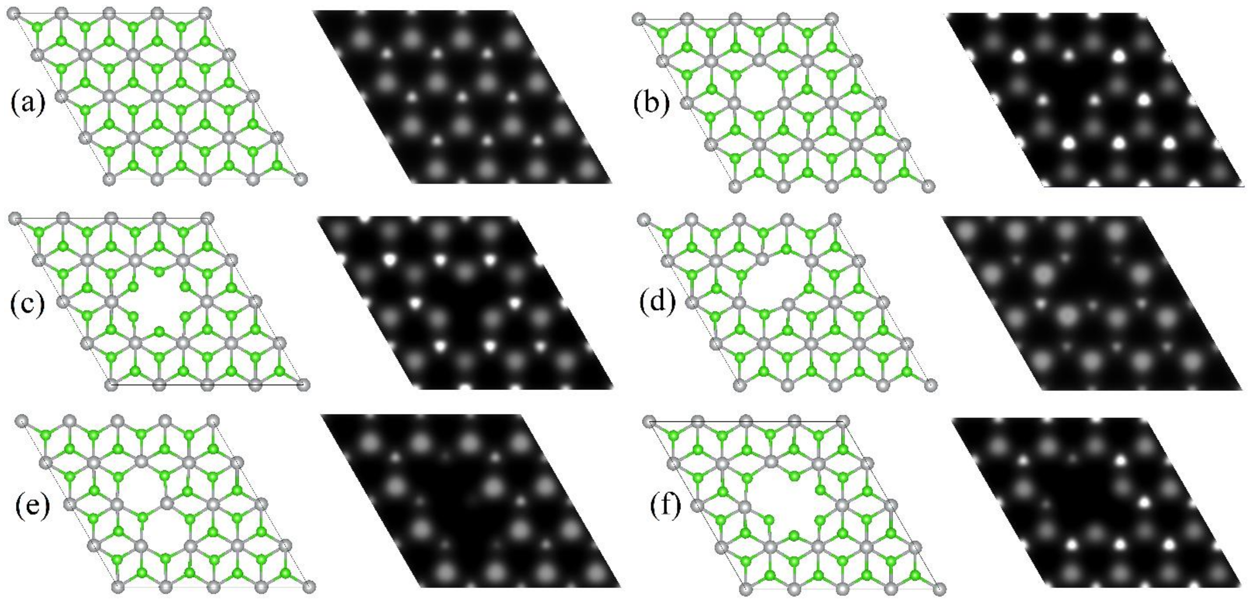

Figure S8. Atomic structure (the left panels) and STM images at a constant height mode (the right panels) of (a) pure, (b) SV_{Cl}-containing, (c) SV_{M}-containing, (d) DV^{I}_{Cl}-containing, (e) DV^{II}_{Cl}-containing, and (f) DV_{MCl}-containing 2D $NiCl_2$.

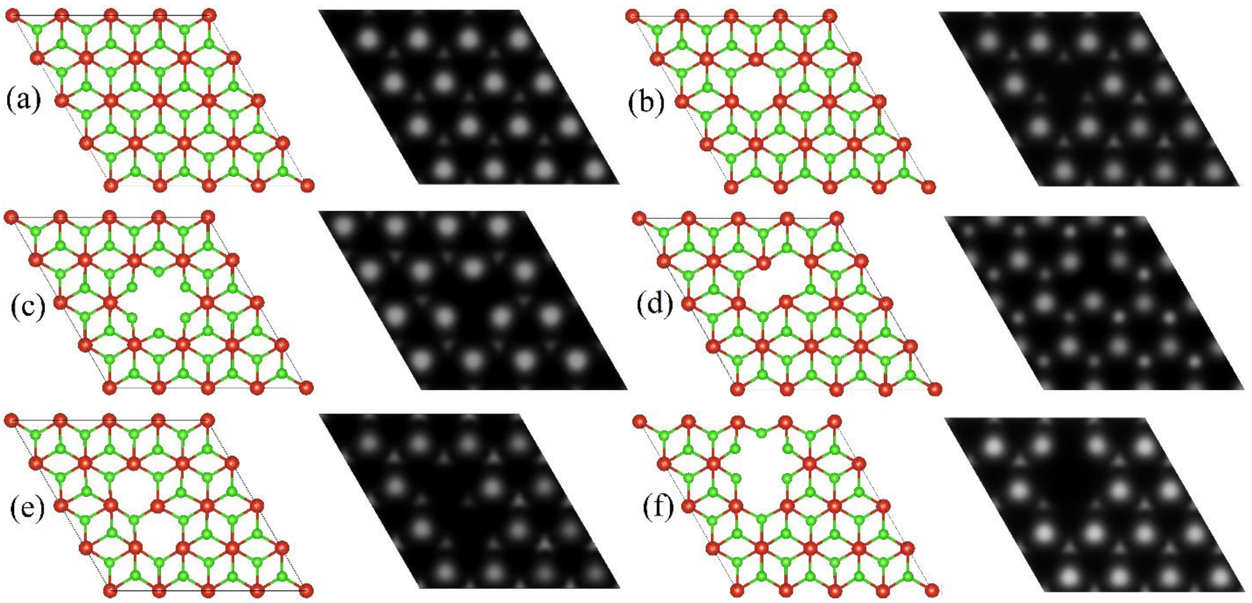

Figure S9. Atomic structure (the left panels) and STM images at a constant height mode (the right panels) of (a) pure, (b) SV_{Cl}-containing, (c) SV_{M}-containing, (d) DV^{I}_{Cl}-containing, (e) DV^{II}_{Cl}-containing, and (f) DV_{MCl}-containing 2D VCl_2.

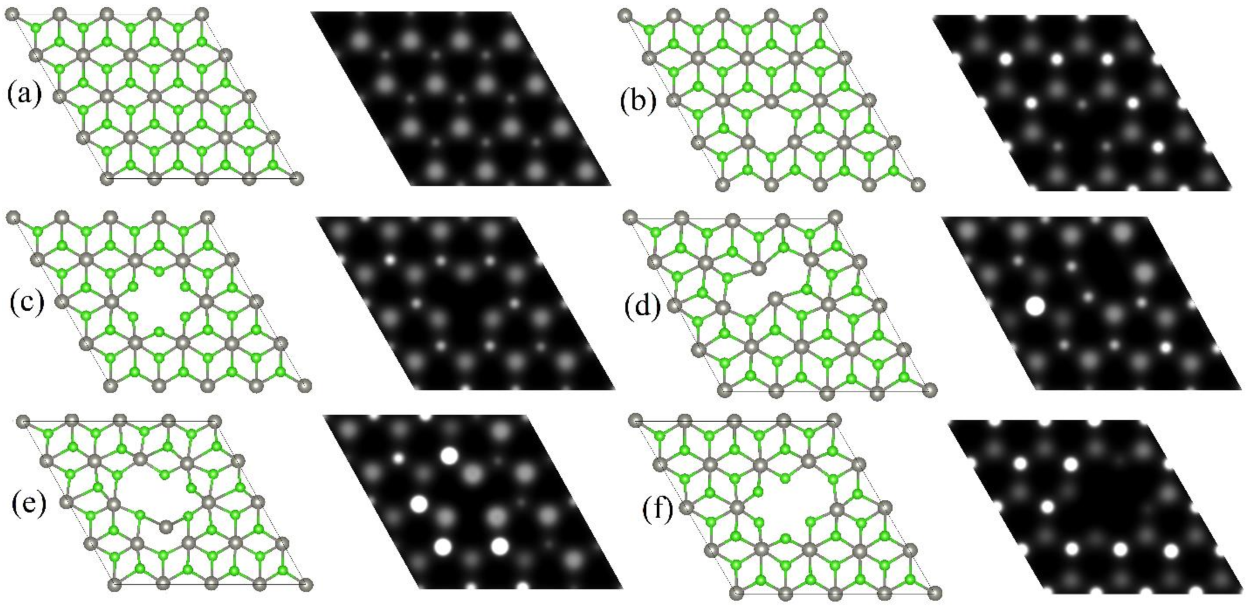

Figure S10. Atomic structure (the left panels) and STM images at a constant height mode (the right panels) of (a) pure, (b) SV_{Cl}-containing, (c) SV_{M}-containing, (d) DV^{I}_{Cl}-containing, (e) DV^{II}_{Cl}-containing, and (f) DV_{MCl}-containing 2D $ZnCl_2$.

Section 7. H_2O and O_2 absorption on 2D MCl_2

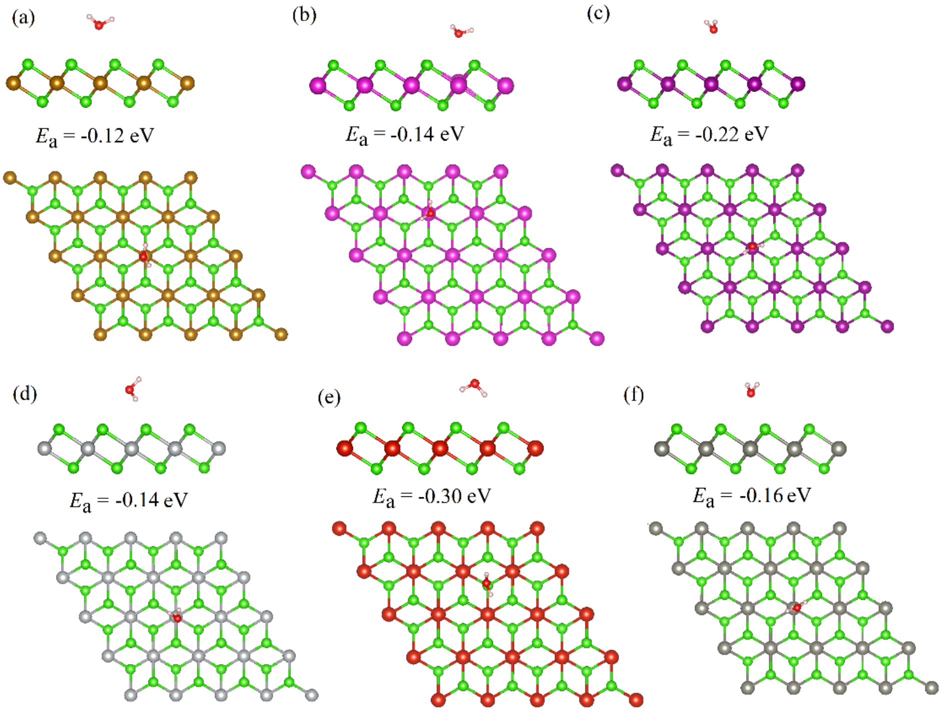

Figure S11. Top and side view of atomic structure of the H_2O molecule absorbed on (a) 2D $FeCl_2$, (b) 2D $CdCl_2$, (c) 2D $MnCl_2$, (d) 2D $NiCl_2$, (e) 2D VCl_2, and (f) 2D $ZnCl_2$.

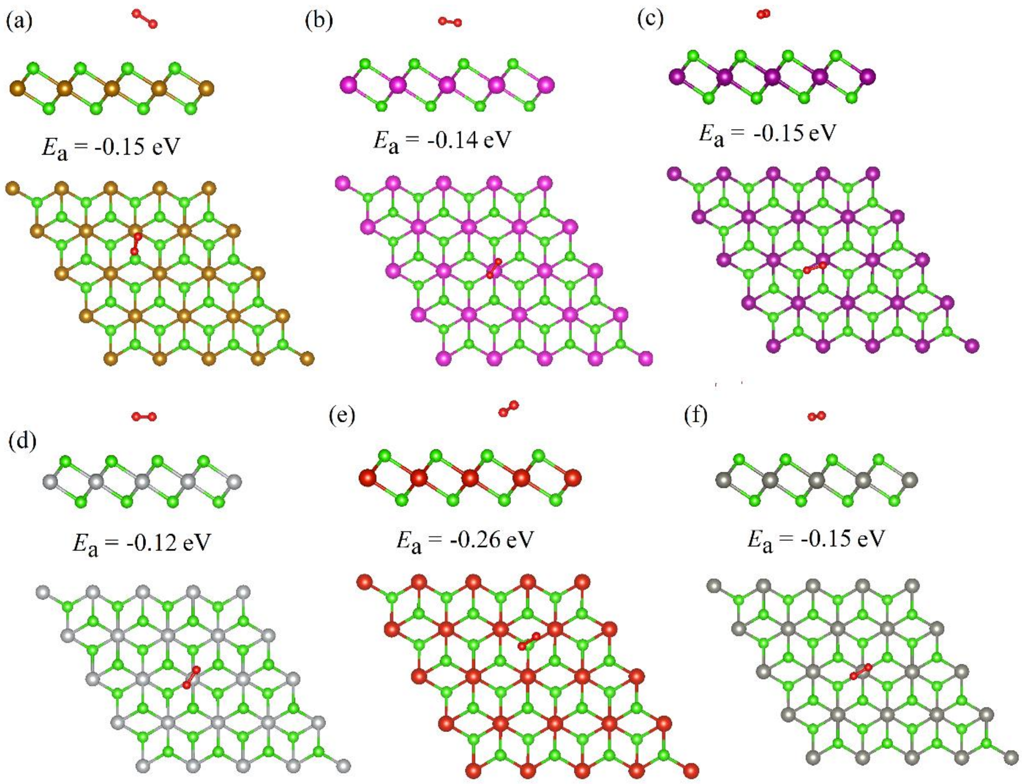

Figure S12. Top and side view of atomic structure of the O_2 molecule absorbed on (a) 2D $FeCl_2$, (b) 2D $CdCl_2$, (c) 2D $MnCl_2$, (d) 2D $NiCl_2$, (e) 2D VCl_2, and (f) 2D $ZnCl_2$.

Table S3. *Comparison of E_a of H_2O and O_2 on* 2DMCl_2 and other common 2D materials.

	E_a of H_2O, eV	*E_a of O_2*, eV
2D $FeCl_2$	-0.12	-0.15
2D $CdCl_2$	-0.14	-0.14
2D $MnCl_2$	-0.22	-0.15
2D $NiCl_2$	-0.14	-0.12
2D VCl_2	-0.30	-0.26
2D $ZnCl_2$	-0.16	-0.15
Graphene[51]	-0.27	-0.04
Phosphorene[49]	-0.14	-0.27
Arsenene[49]	-0.19	-0.54
Antimonene[49]	-0.20	-0.61
Bismuthene[49]	-0.14	-0.61
2D phosphorus carbide[51]	-0.24	-0.59
